# γδ T Cells May Aggravate Acute Graft-Versus-Host Disease Through CXCR4 Signaling After Allogeneic Hematopoietic Transplantation

**DOI:** 10.3389/fimmu.2021.687961

**Published:** 2021-07-14

**Authors:** Ning Wu, Ruoyang Liu, Shuang Liang, Haitao Gao, Lan-Ping Xu, Xiao-Hui Zhang, Jiangying Liu, Xiao-Jun Huang

**Affiliations:** ^1^ Peking University People’s Hospital, Peking University Institute of Hematology, National Clinical Research Center for Hematologic Disease, Beijing Key Laboratory of Hematopoietic Stem Cell Transplantation, Beijing, China; ^2^ Nanfang Hospital, Southern Medical University, Guangzhou, China; ^3^ Peking-Tsinghua Center for Life Sciences, Academy for Advanced Interdisciplinary Studies, Peking University, Beijing, China

**Keywords:** acute graft-versus-host disease, γδ T cells, CXCR4, T cell migration, hematopoietic cell transplantation

## Abstract

Graft-versus-host disease (GVHD) is a pathology in which chemokines and their receptors play essential roles in directing the migration of alloreactive donor T cells into GVHD organs, thereby leading to further target tissue damage. Currently, acute GVHD (aGVHD) remains a major cause of high morbidity and mortality in patients who underwent allogeneic hematopoietic cell transplantation (alloHCT). The identification of immune cells that correlate with aGVHD is important and intriguing. To date, the involvement of innate-like γδ T cells in the pathogenesis of aGVHD is unclear. Herein, we found that primary human γδ T cells did not directly trigger allogeneic reactions. Instead, we revealed that γδ T cells facilitated the migration of CD4 T cells *via* the SDF-1-CXCR4 axis. These results indicate indirect regulation of γδ T cells in the development of aGVHD rather than a direct mechanism. Furthermore, we showed that the expression of CXCR4 was significantly elevated in γδ T cells and CD4 and CD8 T cells in recipients who experienced grades II-IV aGVHD after alloHCT. Consistently, CXCR4-expressing γδ T cells and CD4 T cells were induced in the target organs of mice suffering aGVHD. The depletion of γδ T cells in transplant grafts and treatment with AMD3100, an inhibitor of CXCR4 signaling, delayed the onset of aGVHD and prolonged survival in mice. Taken together, these findings suggest a role for γδ T cells in recruiting alloreactive CD4 T cells to target tissues through the expression of CXCR4. Our findings may help in understanding the mechanism of aGVHD and provide novel therapeutic targets.

## Introduction

Acute graft-versus-host disease (aGVHD) remains a leading cause of unfavorable and life-threatening outcomes after allogeneic hematopoietic cell transplantation (alloHCT) (1). The stages of aGVHD are defined based on clinical manifestations and the severity of organ involvement: grade I (mild), II (moderate), III (severe), and IV (very severe). Among patients who underwent alloHCT with different protocols, 30%-50% developed aGVHD (grades I-IV), and 14% experienced severe aGVHD (grades III-IV) (2). Mortality is higher in patients suffering moderate to severe aGVHD than in those with mild disease. Only 25-30% of patients with grade III aGVHD and 1-2% of patients with grade IV aGVHD survived longterm (>2 years) (3). Although prophylaxis and treatment regimens have improved over the past two decades, severe aGVHD after alloHCT is still a challenging problem in the clinic. Therefore, a better understanding of the mechanisms related to the pathogenesis of aGVHD is essential and urgent for decreasing the incidence of this intractable complication and improving therapeutic efficacy.

Currently, the development of aGVHD can be separated into four pathophysiological stages. The first stage centers upon the effects of conditioning regimens before alloHCT and the consequent proinflammatory microenvironment that lays the foundation for initial leukocyte activation and infiltration. The second stage is mainly focused on the activation of T cells. Donor-derived T cells recognize alloantigens on host-derived antigen-presenting cells, resulting in the proliferation and differentiation of alloreactive effector T cells. Then, the activated allogeneic T cells migrate towards target organs. Further release of inflammatory cytokines, accompanied by direct cell-mediated cytotoxicity, leads to the third and final phases of aGVHD (4). During this process, CD4 and CD8 T cells are responsible for mediating the systemic Th1-type response, as well as an inflammatory cytokine cascade (5). Apart from adaptive αβ T cells, the migration and function of innate-like γδ T cells associated with alloreactivity have been less well reported. Although previous studies demonstrated that γδ T cells were involved in the development of aGVHD, the conclusions remain unclear (6–9).

The recruitment of lymphocytes into target tissues is critical for the pathogenesis of aGVHD (10). It is well understood that the migration and infiltration of T cells into specific organs are regulated by a series of chemokines *via* interactions with specific receptors on T cells (11). As revealed by clinical and experimental studies, CCR5 is a critical receptor associated with aGVHD development. CCR5 appears to play a major role in recruiting lymphocytes to the skin and leads to the production of cytokines, which are involved in the pathogenesis of aGVHD (12). Notably, it has been reported that CCR5 is colocalized and forms complexes with the chemokine receptor CXCR4 on the surface of CD4-expressing T cells. CXCR4 interacts with stromal-derived factor-1 (SDF-1/CXCL12) and regulates hematopoietic stem and progenitor cell trafficking (13). AMD3100, an antagonist of CXCR4 signaling, has been proven to act as an immunomodulator to regulate various immune responses, such as mobilizing hematopoietic stem cells, increasing circulating neutrophils, lymphocytes and monocytes, reducing myeloid-derived suppressor cells, and enhancing cytotoxic T cell infiltration in tumors (14). Although the SDF-1-CXCR4 signaling pathway has gained increasing attention in recent years, its role has not been described in association with the development of aGVHD.

In the current study, we examined whether γδ T cells functioned directly or indirectly in the process of alloreactivity and contributed to the development of aGVHD after alloHCT. The involvement of CXCR4 expression and signaling in the association of γδ T cells with aGVHD was also explored. Our findings may help researchers understand the mechanisms behind the occurrence of aGVHD.

## Materials and Methods

### Mixed Lymphocyte Reaction

Peripheral blood samples were collected from healthy donors. Then peripheral blood mononuclear cells (PBMCs) were freshly isolated by Ficoll-Hypaque density gradient centrifugation. Primary γδ T cells and PBMCs depleted of γδ T cells (referred to as PBMCΔγδ T cells) were sorted using Anti-TCR γ/δ MicroBead Kit (Miltenyi Biotec, Germany). After that, the purified allogenic γδ T cells and PBMCΔγδ T cells were resuspended in RPMI 1640 medium plus 10% FBS, followed by coculture crosswise at a 1:3 rate in 96-well round bottom plates (Corning Costar, USA). On day 5 after coculture cells were collected and detected by flow cytometry.

### Detection of Intracellular Cytokines

For detection of intracellular cytokines, cocultured cells were treated with 1x Protein Transport Inhibitor Cocktail (500x, eBioscience, USA) for 5 h at 37°C. Following staining with anti-CD4, anti-CD8 and anti-TCRαβ, cells were fixed and permeabilized with the FIX&PERM kit (MultiSciences Biotech, China), and then were stained with anti-TNF-α, anti-IFN-γ and anti-IL-17 antibodies. Besides, positive controls were included to demonstrate that the cytokine staining was appropriate for use in our study. PBMCs were freshly isolated from healthy donors and treated with Cell Stimulation Cocktail (plus protein transport inhibitors) (500x, eBioscience, USA) for 5 h and collected for detection of intracellular cytokines.

### Cell Migration Assay

The migration assay was designed using Transwell^®^ cell culture plate (Corning Costar, USA). Primary γδ T cells were isolated as described above. Primary αβ T cells were sorted from fresh PBMCs of different healthy donors using Anti-TCR γ/δ MicroBead Kit and Anti-CD3 MicroBead Kit (Miltenyi Biotec, Germany). The purified αβ T cells (5×10^5^ cells) with or without γδ T cells (1.5×10^5^ cells) were placed into the upper chamber with 100 μl of RPMI 1640 culture medium (plus 10% FBS), and 600 μl of RPMI 1640 culture medium containing SDF-1 (100 ng/ml; Biovision) was added to the lower chamber. After incubation at 37°C for 18 h, the cells that had migrated into the lower wells were collected and detected by flow cytometry. The ratio of migrating cells was calculated by dividing the number of cells in the lower chamber by the total input cells, which were delivered onto the upper chamber before migration.

For chemotaxis inhibition assay, primary γδ T cells were positively sorted using anti-TCR γ/δ MicroBead Kit (Miltenyi Biotec, Germany). The purity of positive-selected γδ T cells was over 95%. Then the purified γδ T cells were placed into the upper chamber with 100 μl of RPMI 1640 plus 10% FBS culture medium, and 600 μl of RPMI 1640 culture medium containing SDF-1 (100 ng/ml; Biovision) was added to the lower chamber. After incubation for 24 h, γδ T cells those migrated towards SDF-1 were collected and counted. Then SDF-driven γδ T cells (5×10^5^ cells) were placed onto the lower chambers with or without AMD3100 (10 µg/ml; Selleck) respectively. The non-treated primary γδ T cells were placed onto the lower chamber as control. The upper chambers were each added with the sorted αβ T cells (5×10^5^ cells). Following incubation for 18 h, cells in lower chambers were collected for counting and then detected by flow cytometry. The calculation of CD4 and CD8 T cells mobility is same as described above.

To validate if SDF-1-exposed γδ T cells produce additional SDF-1, primary γδ T cells were sorted and resuspended in culture media with 100, 150, and 200 ng/mL of SDF-1. The non-treated primary γδ T cells and media containing SDF-1 were as controls. After incubation for 24 h, the cell supernatant was collected for subsequent ELISA.

### Patients

The current study included 46 consecutive patients who were diagnosed with hematopoietic malignancies (including acute myeloid leukemia, acute lymphoblastic leukemia, chronic myeloid leukemia, and myelodysplastic syndrome) and received haploidentical hematopoietic cell transplantation (haploHCT) at our institute from September 2018 to December 2018. The included patients were between the ages of 16 and 58. The pre-transplant conditioning regimen, transplant procedures, aGVHD prophylaxis and treatment were described previously (15, 16). Chimerism analyses were performed by DNA fingerprinting of short tandem repeats using peripheral blood samples. In the current study, full donor chimerism was confirmed in all included patients at 30 days after transplantation. Protocol of this study has been approved by the Ethics Committee of Peking University Institute of Hematology. All recipients and donors signed consent forms.

### Flow Cytometry Analyses

Peripheral blood samples were collected from the included patients around 45 days post-haploHCT. Briefly, 300 μl of fresh peripheral blood per sample was stained with the following fluorochrome-labelled antibodies: PE-Cy7-CD3, BV510-TCRαβ, BV605-CD4, APC-CD8, PE-CF594-CXCR4 (BioLegend, San Diego, CA). After incubation, red blood cells were lysed with a lysis solution (BD Biosciences, San Jose, CA) and then were washed twice with PBS. Polychromatic flow cytometric analyses were performed on a BD LSRFortessaTM Cell Analyser and further analyzed using BD FACSDivaTM software.

### Mice

The severe immunodeficient NOD-Prkdc^scid^ Il2rg^tm1^/Vst (NPG) mice were purchased from Beijing Vitalstar Biotechnology (China). For transplantation of human blood cells, NPG mice were irradiated at a dose of 1 Gy. Then PBMCs freshly isolated from healthy donors were injected into the mice (5×10^6^ cells/mouse) *via* the tail vein. Since the second week after injection, the PB samples were collected from mice and the implantation was detected by flow cytometry with APC-Cy7-human CD45 antibody (BioLegend). The implantation will be confirmed if the percentage of human CD45-positive cells in mouse peripheral mononuclear cells is more than 25%.

Mice were graded every 2 days for evidence of GVHD by assessment of five clinical parameters: weight loss, posture (hunching), activity, fur texture, and skin integrity. Individual mouse from coded cages received a score of 0 to 2 for each criterium (maximum score of 10). Scores > 5 were classified as severe GVHD, and 3-5 were classified as moderate GVHD. Mice were sacrificed when they reached final aGVHD stages (weight loss > 25%, severe hunching, impaired movement, stationary unless stimulated, severe ruffling/poor grooming, obvious areas of denuded skin) (17). Given the fact that naive NPG mice would not exhibit GVHD if they were not infused with PBMCs, and the onset time of GVHD in PBMC-infused mice would be varied due to individual differences, when the mice manifested obvious GVHD symptoms, the equal number of mice without aGVHD were sacrificed at the same time and used as the no-GVHD controls. Meanwhile, liver biopsies were retrieved from mice with and without aGVHD. Histolopathological features were evaluated based on hematoxylin & eosin (H&E) staining for formalin-fixed paraffin-embedded (FFPE) sections. Immunophenotypic analyses were conducted on FFPE sections using antibodies against human TNF-α (Boster, China), TCRγδ (Biolegend, USA), CD4 (Affinity Biosciences, China) and CXCR4 (Biosynthesis Biotechnology, China).

The biopsy specimens of skin and liver were obtained from mice with and without aGVHD. Skin cells were gained by mincing the tissues followed by digestion with 1% type 1 collagenase for 4 h. Liver samples were gently ground in PBS to isolate the primary cells. All collected tissue cells were washed twice with PBS, followed by staining with fluorochrome-labelled antibodies as indicated. Polychromatic flow cytometric analyses were performed on a BD LSRFortessaTM Cell Analyser and further analyzed using BD FACSDivaTM software.

To further prove that the γδ T cells and CXCR4 signaling contribute to the migration of CD4 T cells and the development of GVHD, NPG mice were transplanted and divided into four groups: PBMCs group; PBMCs plus AMD3100 group; PBMCs depleted of γδ T cells (PBMCΔγδ T cells) group; and PBMCΔγδ T cells plus AMD3100 group. PBMCs and PBMCΔγδ T cells were injected into the mice respectively as described above. The efficiency of γδ T depletion from PBMCs using Miltenyi kit were almost 98%. Due to the limited number of human blood cells injected into mice, we divided the mice into two batches. In the first batch of mice, AMD3100 was administered intraperitoneally to mice at a dose of 5mg/kg on the next day after injection of human blood cells. In the second batch of mice, we performed multiple injections of AMD3100 (from 1 to 4 times) in mice infused with human blood cells to better observe the survival of mice among each group in consideration of the short half-life of AMD3100. Mice were routinely graded for the signs of aGVHD. The onset time of severe aGVHD and survival days were recorded. When mice died of severe aGVHD, mononuclear cells of skin and liver were obtained and detected by polychromatic flow cytometry as described above. Meanwhile, liver biopsies were retrieved from mice with and without aGVHD. Histolopathological features were evaluated based on H&E staining for FFPE sections.

### Statistical Analysis

The results shown in the current study are representative data from independent experiments, which has been repeated at least three times. Statistical analyses were performed using SPSS 22.0 statistical software (SPSS Inc, USA). Differences in patient age, numbers of mononuclear cells (MNCs) and CD34^+^ cells in transplantation grafts, and the proportions of CXCR4^+^γδ T cells, CXCR4^+^CD4 T cells and CXCR4^+^CD8 T between recipients with 0-I and II-IV aGVHD were compared using the Mann-Whitney U test. Differences in gender and type of primary disease were analyzed using the χ^2^ test. The difference for survival was determined with Kaplan-Meier log-rank test. Statistical significance was defined as *P* ≤ 0.05, based on a two-tailed test.

## Results

### Homeostatic γδ T Cells Neither Responded to Allogeneic T Cell Stimulation nor Induced CD4 and CD8 T Cell Alloreactivity

**Figure 1:**
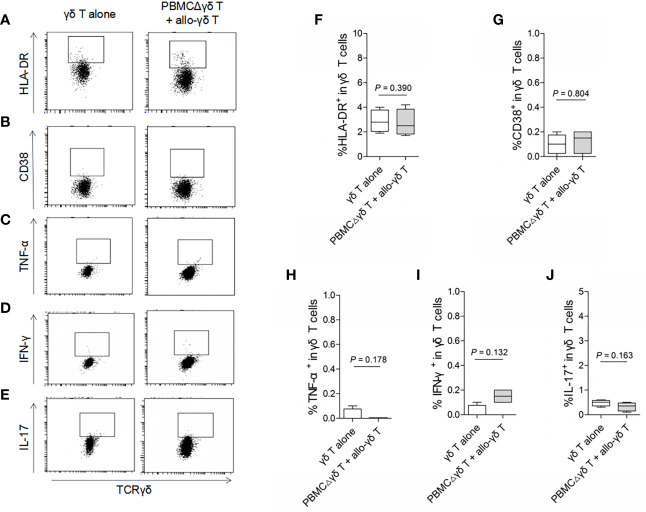
Assay for the reactivity of γδ T cells in response to stimulation of allogeneic αβ T cells. Mixed lymphocyte reactions (MLRs) were performed by co-culture of purified allogenic γδ T cells with PBMCs depleted of γδ T cells (PBMCΔγδ T cells) isolated from healthy donors, at a ratio of 1:3. Cells were collected on day 5 after co-culture and stained with antibodies against γδ TCR **(A–J)**, HLA-DR **(A, F)**, CD38 **(B, G)**, TNF-α **(C, H)**, IFN-γ **(D, I)** and IL-17 **(E, J)**. Data are expressed as the mean ± SD of triplicate cultures. *P* values are shown on the graphs.

To determine whether γδ T cells themselves respond to allogeneic antigen stimulation, a mixed lymphocyte reaction (MLR) experiment was conducted. Purified homeostatic γδ T cells were cocultured with allogeneic PBMCs that had been depleted of γδ T cells (referred to as PBMCΔγδ T cells) from healthy donors. As shown in **Figure 1**, PBMCΔγδ T cells did not increase the expression of the T cell activation markers HLA-DR (**Figures 1A, F**, *P* > 0.05) or CD38 (**Figures 1B, G**, *P* > 0.05) on allogenic γδ T cells compared with the control. In addition, we measured the expression of inflammatory cytokines that are commonly induced by allogeneic reactions. There were no significant differences in the production of TNF-α (**Figures 1C, H**), IFN-γ (**Figures 1D, I**) and IL-17 (**Figures 1E, J**) by allogenic γδ T cells in the MLR and control groups (all *P* > 0.05).

**Figure 2:**
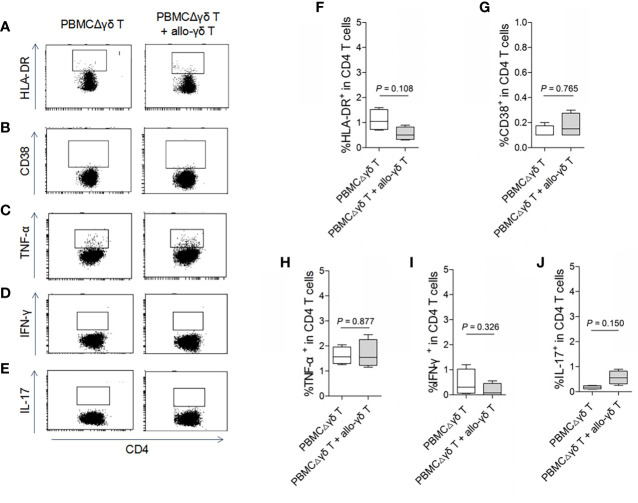
Assay for the reactivity of CD4 T cells in response to stimulation of allogeneic γδ T cells. MLRs were performed by co-culture of purified allogenic γδ T cells with PBMCΔγδ T cells isolated from healthy donors, at a ratio of 1:3. Cells were collected on day 5 after co-culture and stained with antibodies against CD4 **(A–J)**, HLA-DR **(A, F)**, CD38 **(B, G)**, TNF-α **(C, H)**, IFN-γ **(D, I)** and IL-17 **(E, J)**. Data are expressed as the mean ± SD of triplicate cultures. *P* values are shown on the graphs.

**Figure 3:**
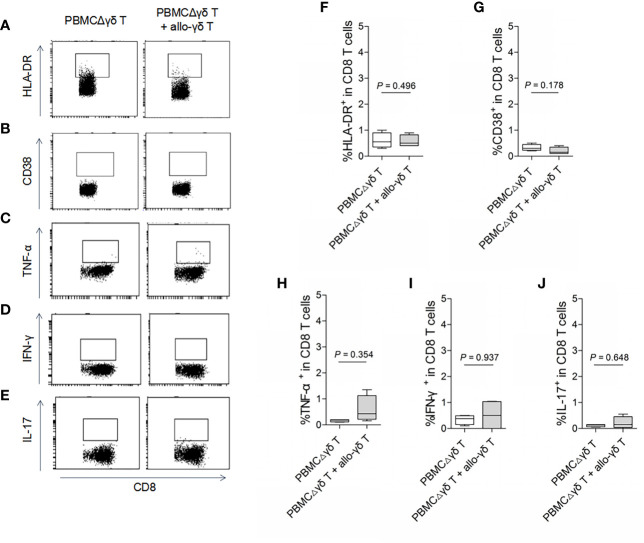
Assay for the reactivity of CD8 T cells in response to stimulation of allogeneic γδ T cells. MLRs were performed by co-culture of purified allogenic γδ T cells with PBMCΔγδ T cells isolated from healthy donors, at a ratio of 1:3. Cells were collected on day 5 after co-culture and stained with antibodies against CD8 **(A–J)**, HLA-DR **(A, F)**, CD38 **(B, G)**, TNF-α **(C, H)**, IFN-γ **(D, I)** and IL-17 **(E, J)**. Data are expressed as the mean ± SD of triplicate cultures. *P* values are shown on the graphs.

On the other hand, we examined whether γδ T cells directly stimulated allogeneic responses. As we hypothesized, coculture with allogenic γδ T cells did not increase the expression of HLA-DR or CD38 on either CD4 (**Figures 2A, B, F, G**, *P* > 0.05) or CD8 T cells (**Figures 3A, B, F, G**, *P* > 0.05) compared with those in the control groups. Similarly, the production of the inflammatory cytokines TNF-α (**Figures 2C, H** and **3C, H**), IFN-γ (**Figures 2D, I** and **3D, I**) and IL-17 (**Figures 2E, J** and **3E, J**) by CD4 and CD8 T cells was not significantly different between the MLR and control groups (all *P* > 0.05). In contrast, the production of TNF-α, IFN-γ and IL-17 by γδ T, CD4 and CD8 T cells was significantly increased after stimulation with a pan-T cell stimulator, as shown in **Supplementary Figures 1–3** (all *P* < 0.05). These results indicated that cytokine staining was appropriate for use in our study. Taken together, these results showed that primary homeostatic γδ T cells could not directly trigger allogeneic reactions.

### γδ T Cells Promoted the Migration of CD4 T Cells Through the SDF-1-CXCR4 Axis

**Figure 4:**
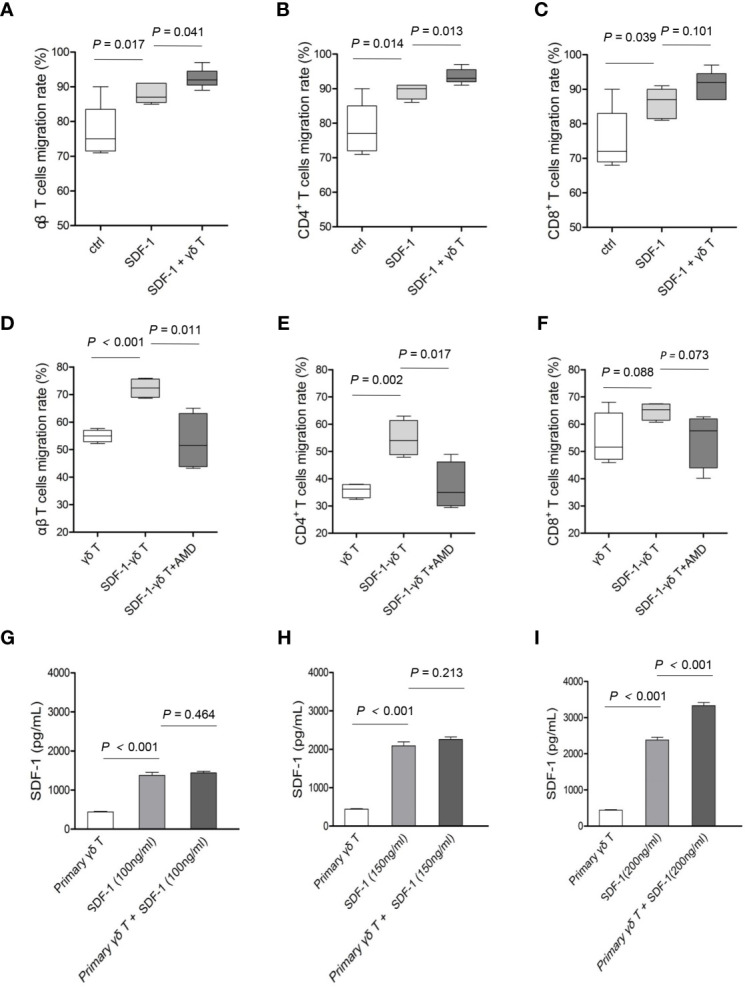
The impact of γδ T cells and SDF-1-CXCR4 axis on the migrations of CD4 and CD8 T cells. The migratory abilities of αβ T cells **(A)**, CD4 T cells **(B)** and CD8 T cells **(C)** towards γδ T cells were examined, with or without SDF-1. The migratory abilities of αβ T cells **(D)**, CD4 T cells **(E)** and CD8 T cells **(F)** towards SDF-1-driven γδ T cells were examined, with or without an antagonist (AMD3100) of CXCR4 signaling. The supernatants of γδ T cells pretreated with indicated concentrations of SDF-1 for 24 h were collected for ELISA test **(G–I).** Data are expressed as the mean ± SD of triplicate cultures. *P* values are shown on the graphs.

Next, we examined whether γδ T cells played an indirect role in allogeneic reactions by promoting the migration of αβ T cells. In the presence of the CXCR4 ligand SDF-1, the migration of total αβ T cells (**Figure 4A**, *P* = 0.017) and CD4 T cells (**Figure 4B**, *P* = 0.014) was increased compared with that of the control groups without SDF-1. The addition of autologous γδ T cells into the culture system further promoted the migration of total αβ T cells (**Figure 4A**, *P* = 0.041) and CD4 T cells (**Figure 4B**, *P* = 0.013) towards SDF-1. Although more CD8 T cells migrated towards SDF-1 than in the control group (**Figure 4C**, *P* = 0.039), these cells were not further increased in the presence of γδ T cells (**Figure 4C**, *P* = 0.101).

To confirm that γδ T cells are capable of promoting the migration of CD4 T cells through the SDF-1-CXCR4 axis, γδ T cells that had premigrated towards SDF-1 (referred to as SDF-1-γδ T cells) were used in the following chemotaxis assay with or without AMD3100, an inhibitor of the SDF-1-CXCR4 signaling pathway. As shown in **Figure 4**, the migration rates of total αβ T cells (**Figure 4D**, *P* < 0.001) and CD4 T cells (**Figure 4E**, *P* = 0.002) were markedly increased when SDF-1-γδ T cells were added into the lower chamber compared with those in the presence of untreated γδ T cells. In contrast, AMD3100 significantly attenuated the migration of total αβ T cells (**Figure 4D**, *P* = 0.011) and CD4 T cells (**Figure 4E**, *P* = 0.017) in response to SDF-1-γδ T cells. However, there was no significant change in the migration of CD8 T cells towards SDF-1-γδ T cells, with or without AMD3100 (**Figure 4F**, *P* values are indicated on the graph). Moreover, we found that the secretion of SDF-1 by γδ T cells exposed to different concentrations of exogenous SDF-1 was significantly increased in response to a higher dose of extrinsic SDF-1 (**Figures 4G–I**, *P* values are indicated on the graph). These results suggest that CXCR4-expressing γδ T cells interact with SDF-1 and accelerate the migration of autologous CD4 T cells by producing additional SDF-1.

### CXCR4 Expression Was Increased in γδ T Cells and αβ T Cells in Recipients Who Experienced Grades II-IV aGVHD After alloHCT

Since T cell migration plays an important role in the development of aGVHD, we next investigated the proportions of CXCR4-positive T cells in recipients with or without aGVHD after alloHCT. The clinical characteristics of the patients in the current study are summarized in **Table 1**. There were no significant differences in age, sex, types of disease at diagnosis, or numbers of MNCs and CD34^+^ cells in the grafts between recipients with grades 0-I and II-IV aGVHD after haploHCT (all *P* > 0.05). The conditioning regimen, transplantation procedure, and aGVHD prophylaxis and treatment regimens were the same for all included patients. Among 46 patients, 36 (78.3%) were diagnosed with grades 0-I aGVHD, whereas 10 (21.7%) patients developed grades II-IV aGVHD. The median onset time of grades II-IV aGVHD was day 19 after transplantation.

**Table 1:** Clinical characteristics.

	aGVHD 0-I	aGVHD II-IV	*P* value
Subjects, *n*	36	10	–
Age, years	36 (18-58)	26 (19-55)	0.410
Gender, *n* (%)			0.211
Male	19 (53)	3 (30)	
Female	17 (47)	7 (70)	
Diagnosis, *n* (%)			0.546
AML	12 (33)	2 (20)	
ALL	16 (44)	7 (70)	
CML	1 (2.8)	1 (10)	
MDS	5 (14)	0 (0)	
others	2 (5.6)	0 (0)	
Donor type	Related donor	Related donor	–
HLA typing	1-3/6 mismatch	1-3/6 mismatch	–
Stem cell source	BM+PB	BM+PB	–
MNC in grafts,10^8^ /kg	8 (4-15)	9 (7-16)	0.213
CD34^+^ cells in grafts,10^6^ /kg	2 (1-8)	2 (1-7)	0.946
Conditioning regimen	BU+CY+ATG	BU+CY+ATG	–
GVHD prophylaxis	CsA+MMF+MTX	CsA+MMF+MTX	–

ATG, antithymocyte globulin; AML, acute myeloid leukemia; ALL, acute lymphoblastic leukemia; BM, bone marrow; BU, busulfan; CML, chronic myeloid leukemia; CsA, cyclosporine A; CY, cyclophosphamide; MDS, myelodysplastic syndrome; MMF, mycophenolate mofetil; MTX, methotrexate; PB, peripheral blood.

**Figure 5:**
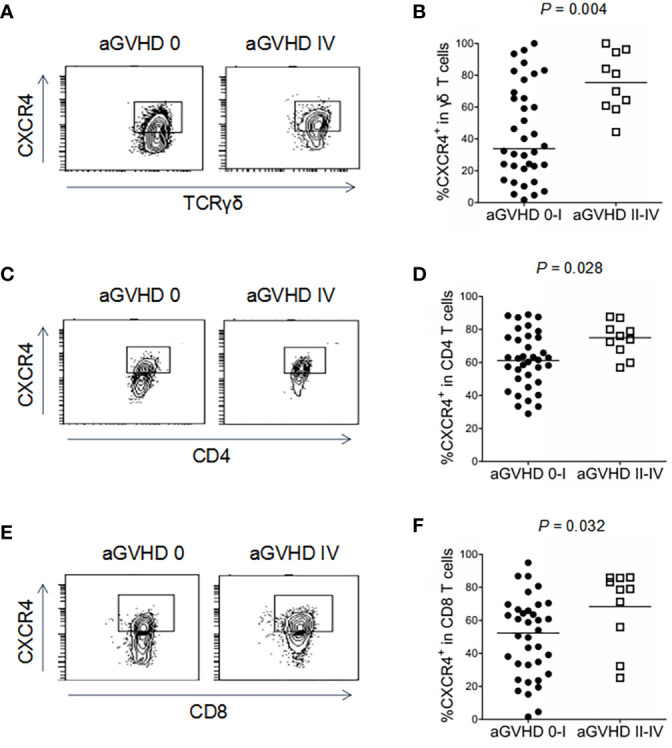
Association of CXCR4 expression in γδ T cells, CD4 and CD8 T cells with aGVHD in haploidentical HCT recipients. The proportions of CXCR4^+^γδ T cells **(A, B)**, CXCR4^+^CD4 T cells **(C, D)**, and CXCR4^+^CD8 T cells **(E, F)** were detected by flow cytometry in recipients with grade 0-I and II-IV aGVHD after haploHCT. *P* values are shown on the graphs.

Interestingly, the proportion of CXCR4-positive cells among peripheral γδ T cells was significantly elevated in recipients with grades II-IV aGVHD compared with those who developed grades 0-I aGVHD (75.5% versus 34.0%, *P* = 0.004, **Figures 5A, B**) at 45 days after alloHCT. Similarly, significant increases in CXCR4 expression were found in CD4 (*P* = 0.028, **Figures 5C, D**) and CD8 (*P* = 0.032, **Figures 5E, F**) T cells in recipients with grades II-IV aGVHD compared with those who developed grades 0-I aGVHD. The gating strategy for the positive populations shown in **Figures 5A, C, E** were based on control cells without any staining and cells that were stained with all antibodies minus CXCR4 (**Supplementary Figure 4**). Additionally, the CD4 and CD8 cells in the current study referred to conventional CD4^+^ and CD8^+^ αβ T cells. In addition to γδ T cells, these are major subpopulations of effector T cells. Although some γδ T cells express CD8, the proportion was only approximately 8% in our unpublished data. Taken together, these results suggest that increased CXCR4 expression on γδ and αβ T cells may positively correlate with the development of aGVHD after alloHCT.

### CXCR4-Expressing γδ and CD4 T Cells Were Increased in the Target Organs of Mice With aGVHD

**Figure 6:**
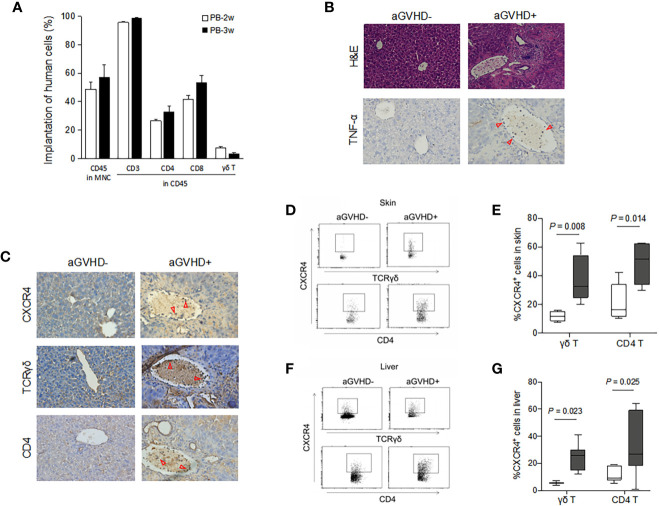
Migrations of human γδ T cells and CD4 T cells expressing CXCR4 in mice with or without aGVHD. Immunodeficient mice were transplanted with human PBMCs as described. The proportion of human CD45-positive cells was detected in mouse peripheral blood by flow cytometry at 2 and 3 weeks after injection **(A)**. Histopathological examination was performed on biopsy specimens of liver obtained from transplanted mice. Histopathological feature and expression of TNF-α in liver were detected by hematoxylin and eosin (H&E, ×100) staining and immunohistochemistry analyses, ×200 **(B)**. Three adjacent sections of liver biopsies were stained with antibodies against CXCR4, TCRγδ and CD4, ×200 **(C)**. CXCR4-expressing γδ T and CD4 T cells were detected by flow cytometry in the skin **(D, E)** and liver **(F, G)** of mice with or without aGVHD. n = 4, *P* values are shown on the graphs.

Immunodeficient mice were injected with human PBMCs isolated from healthy donors. As shown in **Figure 6A**, more than 50% of human CD45-positive cells were observed in mouse peripheral mononuclear cells (49% and 57%) at 2 and 3 weeks after injection, indicating that implantation was successful in this model. The majority (> 95%) of implanted human blood cells were CD3 T cells. CD4, CD8, and γδ T cells were all detectable in the peripheral blood of transplanted mice (**Figure 6A**), and the proportions were close to the normal levels of T cell subsets in healthy subjects (data not shown). H&E staining showed that lymphocytes infiltrated around the portal areas of the livers in aGVHD mice, and these cells were absent in control mice (**Figure 6B**). Lymphocyte infiltration and epithelial disruption were visible in many bile ducts in mice with aGVHD compared to those of mice without aGVHD (**Figure 6B**). The presence and location of the typical inflammatory cytokine TNF-α was observed in aGVHD lesions in the liver, which further indicated that liver inflammatory foci formed during aGVHD (**Figure 6B**).

Next, we evaluated the presence and location of T cell subsets in the target organs of mice suffering aGVHD. Representative images of immunohistochemical staining for the expression of CXCR4, TCRγδ, and CD4 in the liver are shown in **Figure 6C**. CXCR4 protein expression was significantly detectable in lymphocytes infiltrating around the portal areas in the livers of aGVHD mice (**Figure 6C**). In contrast, little CXCR4 was observed throughout the liver parenchyma in control mice. Consistently, there was obvious emergence of γδ T cells and CD4 T cells in defined liver areas in aGVHD mice, and these cells infiltrated the tissue surrounding the portal areas. However, γδ T cells and CD4 T cells were hardly observed in mice without aGVHD. Flow cytometry further confirmed that CXCR4-positive γδ T cells and CXCR4-positive CD4 T cells were significantly elevated in the skin of aGVHD mice compared with control mice (*P* = 0.008 and *P* = 0.014, respectively, **Figures 6D, E**). Similarly, increased expression of CXCR4 on γδ T cells and CD4 T cells was observed in the livers of aGVHD mice compared with control mice (*P* = 0.023 and *P* = 0.025, respectively, **Figures 6F, G**). Taken together, these results suggest that the expression of CXCR4 on γδ T cells and CD4 T cells promotes the migration of these cells into target organs and thereby induces cutaneous and hepatic aGVHD.

### Depletion of γδ T Cells and Treatment With a CXCR4 Signaling Antagonist Decreased the Migration of CD4 T Cells, Attenuated the Severity of aGVHD and Prolonged Survival in Mice

**Figure 7:**
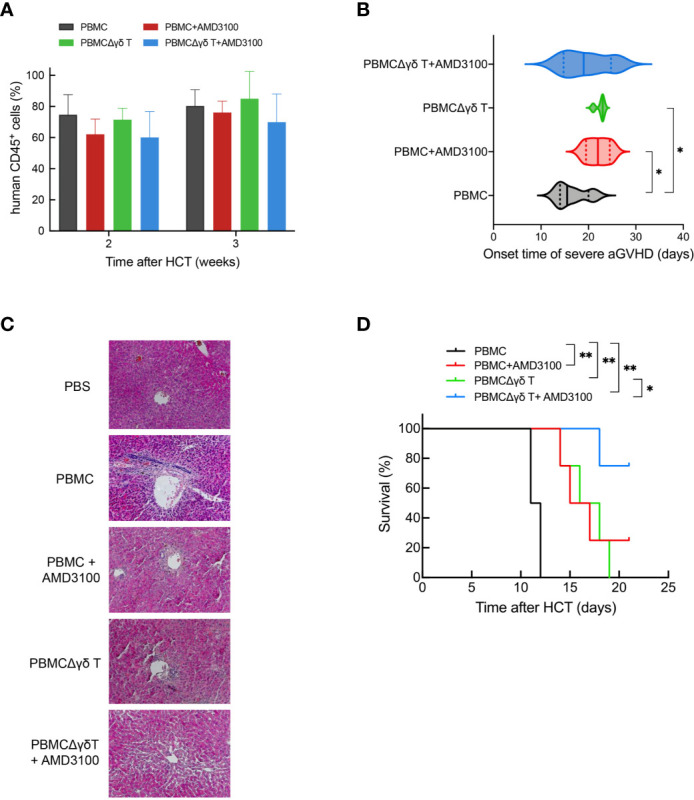
Impacts of γδ T cells and inhibitor of CXCR4 signaling on the onset of severe aGVHD and the survival time in humanized mice. The proportion of human CD45-positive cells was detected by flow cytometry at 2 and 3 weeks in mouse peripheral blood after injection with human PBMCs, PBMCs plus AMD3100, PBMCΔγδ T cells, and PBMCΔγδ T cells plus AMD3100 **(A)**. The onset time of severe aGVHD among each group were depicted **(B)**. Histopathological examination was performed on biopsy specimens of liver obtained from transplanted mice **(C)**. The survival time among each group were depicted **(D)**. n = 4, **P < 0.01, *P < 0.05, other *P* values are shown on the graphs.

To further verify that γδ T cells and CXCR4 signaling are involved in the migration of CD4 T cells and the development of aGVHD, immunodeficient mice were injected with human PBMCs, PBMCs plus AMD3100, PBMCs depleted of γδ T cells (PBMCΔγδ T cells), or PBMCΔγδ T cells plus AMD3100. As shown in **Figure 7A**, the proportion of human CD45-positive cells was more than 60% in the peripheral blood of mice in each group at 2 and 3 weeks after injection. This observation indicated that human lymphocytes were successfully transplanted into the mice. The median onset time of severe aGVHD in mice with human PBMCs was 16.5 (14-21) days after transplantation. In contrast, the occurrence of severe aGVHD was significantly delayed in mice transplanted with PBMCs plus AMD3100 (22, 19-25 days, *P* = 0.040) or mice transplanted with PBMCΔγδ T cells (22.5, 21-23 days, *P* = 0.013, **Figure 7B**). These results indicate that treatment with AMD3100 or the depletion of γδ T cells prolonged the onset of aGVHD in mice transplanted with human blood cells. The median onset time of severe aGVHD in mice with PBMCΔγδ T cells plus AMD3100 (19.5, 14-26 days) was not significantly different from that in the other groups (*P* = 0.368, **Figure 7B**). H&E staining showed that lymphocyte infiltration and epithelial disruption were visible in many bile ducts in the mice that received PBMCs, and this effect was absent in mice that received PBMCs depleted of γδ T cells or treated with the SDF-1-CXCR4 antagonist (**Figure 7C**).

Considering that AMD3100 has a very short half-life and is rapidly eliminated, with a terminal half-life of 5.3 hours, we performed multiple injections of AMD3100 (from 1 to 4 times) in mice infused with human blood cells and observed differences in survival time among each group until the end of experiment (21 days). The control mouse group transplanted with human PBMCs all died due to GVHD around 11.5 (11-12) days. In contrast, the survival time was significantly prolonged in mice transplanted with PBMCs plus AMD3100 (16.8, 14-21 days, *P* = 0.016) or with PBMCΔγδ T cells (16.8, 14-19 days, *P* = 0.004). Additionally, the median survival time of mice transplanted with PBMCΔγδ T cells plus AMD3100 was further extended compared with that in mice transplanted with PBMCs (20.3, 18-21 days, *P* < 0.001, **Figure 7D**). Of note, three of the four mice in this group were still alive at the end of observation. These results highlight the role of γδ T cells in the development of GVHD through CXCR4 signaling.

**Figure 8:**
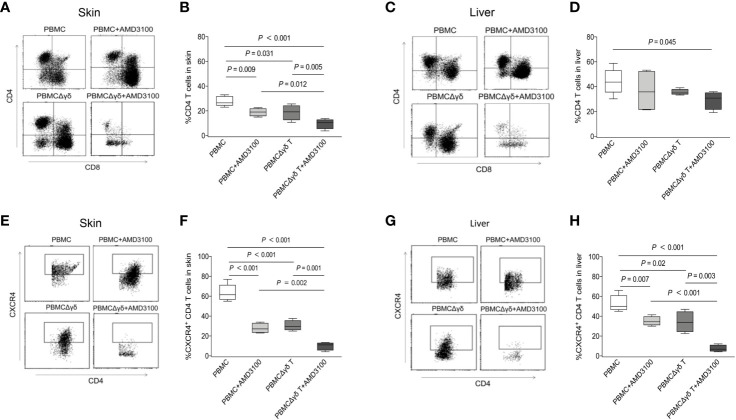
Impacts of γδ T cells and inhibitor of CXCR4 signaling on the migration of CD4 T cells in humanized mice. Presence of CD4 T cells was detected by flow cytometry in the skin **(A, B)** and liver **(C, D) **of mice among each group. CXCR4-expressing CD4 T cells were detected by flow cytometry in the skin **(E, F)** and liver **(G, H)** of mice among each group. n = 4, *P* values are shown on the graphs.

Finally, we examined the migration and localization of CD4 T cells in the target organs of aGVHD in mice after different injections. Flow cytometry showed that 27.8% of CD4 T cells resided in the skin of mice transplanted with PBMCs, and this effect was markedly attenuated in mice administered PBMCs plus AMD3100 (18.9%, *P* = 0.009) or PBMCΔγδ T cells (18.7%, *P* = 0.031). In mice treated with PBMCΔγδ T cells plus AMD3100, the proportion of total CD4 T cells in the skin was further reduced compared with that in the other three groups (9.6%, *P* < 0.001, *P* = 0.012, and *P* = 0.005, respectively, **Figures 8A, B**). As we hypothesized, dramatically higher CXCR4 expression in CD4 T cells was found in the skin of mice that were transplanted with PBMCs than in mice in the PBMC plus AMD3100 and PBMCΔγδ T groups (*P* < 0.001). In parallel, CXCR4-positive CD4 T cells were lowest in the skin of the PBMCΔγδ T cells plus AMD3100 group compared with the other three groups (*P* < 0.001, *P* = 0.002, and *P* = 0.001, respectively, **Figures 8E, F**). The translocation of CD4 T cells in the liver was examined, and the depletion of γδ T cells plus AMD3100 significantly decreased the proportion of total CD4 T cells compared with that in the PBMC group (*P* = 0.045), but this effect was not significantly different from that in the PBMC plus AMD3100 or PBMCΔγδ T cell group (*P* > 0.05, **Figures 8C, D**). Notably, obviously increased expression of CXCR4 on CD4 T cells was observed in the livers of the PBMC group compared with the PBMC plus AMD3100 and PBMCΔγδ T cell groups (*P* = 0.007 and *P* = 0.020). Similar to observations in the skin, PBMCΔγδ T cells plus AMD3100 further reduced the translocation of CXCR4-positive CD4 T cells in the liver compared with that in the other three groups (*P* < 0.001, *P* < 0.001, and *P* = 0.003, respectively, **Figures 8G, H**). Taken together, these results suggest that γδ T cells and CXCR4 signaling significantly contribute to the migration of CD4 T cells into aGVHD target organs. CXCR4 antagonist may prevent γδ T cells from promoting the migration of CD4 T cells through CXCR4 signaling and thereby delay the onset of severe cutaneous and hepatic aGVHD in mice.

## Discussion

In the human immune system, γδ T cells are a unique subpopulation of lymphocytes that mediate various types of immune responses and immunopathological processes (18). It has been extensively reported that γδ T cells play a significant role in host defense against different pathogens, including viruses, bacteria and parasites (19). Moreover, γδ T cells exert antitumor effects against diverse kinds of solid tumors and hematological malignancies (20, 21). Since they function independent of the assistance of MHC molecules, γδ T cells are conventionally recognized not to trigger alloreactivity. Indeed, a previous review showed that selective infusion of donor γδ T cells improved graft-versus leukemia (GVL) effects but did not induce GVHD (22). However, preliminary clinical studies indicated that γδ T cells were associated with aGVHD after HCT. Caroline Pabst et al. found that the absolute number of γδ T cells in the grafts of patients who developed grades II-IV aGVHD was significantly higher than that in patients with grade 0-I aGVHD (23). In contrast, Y Kawanishi et al. conducted clinical studies and showed that γδ T cells were not associated with aGVHD (24). In murine models, the role of γδ T cells in the occurrence of GVHD also remains unclear. Drobyski et al. suggested a protective effect of activated γδ T cells that could mitigate GVHD by modulating the abilities of αβ T cells after bone marrow transplantation (8). However, other studies held an opposite conclusion and showed that the presence of γδ T cells exacerbated lethal GVHD in mice (7, 9). In addition, Anderson et al. did not find any correlation between host γδ T cells and the occurrence of GVHD in mice (6). Such discrepancies in previous studies require clarification. To date, no reports have dissected the role of γδ T cells in the migration of donor T cells towards aGVHD target organs. In the current study, we found that γδ T cells did not directly trigger the allogeneic reactions of CD4 and CD8 T cells. Instead, we observed that the migration of CD4 T cells was significantly affected by γδ T cells through the chemotactic SDF-1-CXCR4 axis. These results suggest that γδ T cells may play an indirect role in promoting the development of aGVHD after transplantation.

Consistently, we found that CXCR4 expression was significantly upregulated in peripheral γδ T cells and CD4 and CD8 T cells in recipients who developed grades II-IV aGVHD after alloHCT. Due to technical limitations, it is impractical to measure the expression of CXCR4 in T cell subsets that migrated into GVHD target organs in transplant patients. Therefore, we established a humanized mouse model to monitor the migration of peripheral effector T cells and the changes in CXCR4 expression in the context of GVHD. Unlike the mouse models used in previous related studies, we utilized severe combined immunodeficient mice with deletion of murine T, B and NK cells and deficient humoral immunity. Therefore, our mouse model had the advantage of simulating clinical immune circumstances after alloHCT. In line with our data from the clinical cohort, CXCR4 expression was remarkably increased in γδ T cells, and CD4 T cells migrated into the target organs of aGVHD mice. Interestingly, although the proportion of CXCR4-positive cells in peripheral CD8 T cells was significantly elevated in recipients with grades II-IV aGVHD compared with those who developed grades 0-I aGVHD, we did not find a significant correlation between CXCR4-positive CD8 T cells and aGVHD in mice (data not shown). One of possible explanations is that the sample size of our murine model was insufficient to reach an explicit result. On the other hand, since CD4 T cells expressed much higher CXCR4 than CD8 T cells, the recruitment of CD4 T cells to target organs of aGVHD mice may rely more heavily on the chemotactic SDF-1-CXCR4 axis than the recruitment of CD8 T cells.

We hypothesized that the recruitment of T cells into aGVHD target organs after alloHCT was mediated at least in part through CXCR4^+^ γδ T cells. Our chemotaxis assay showed that the migration rate of CD4 T cells was apparently increased when γδ T cells were added, especially in the presence of SDF-1-driven γδ T cells. Moreover, the CXCR4 signaling antagonist AMD3100 significantly attenuated the migration of CD4 T cells in the current study. Given that exposure to SDF-1 increased the autocrine secretion of γδ T cells, CXCR4-expressing γδ T cells probably promoted the migration/infiltration of αβ T cells *via* the production of additional SDF-1. Furthermore, we found that depletion of γδ T cells and treatment with the antagonist significantly restrained the migration of CXCR4-expressing CD4 T cells in the target organs, which therefore postponed the onset of severe aGVHD in mice transplanted with human PBMCs. These data indicate a potential role of γδ T cells that express chemokines (e.g., CXCR4) and thereby facilitate CD4 T cell recruitment towards GVHD target organs. As for the possible mechanism by which AMD3100 treatment reduces the severity of GVHD, a previous study demonstrated the efficacy of posttransplant administration of AMD3100 in improving survival using a congenic mouse transplantation model and reasoned that AMD3100 would attenuate the cytokine storm and hence reduce multiorgan toxicities (25). In parallel, another clinical study indicated that grades III–IV acute GVHD was higher in the comparative control cohort than in the plerixafor (AMD3100) treatment group. The plasma MIP-1β level was significantly lower at day +30 in plerixafor-treated patients, supporting the role of CXCR4 antagonism in attenuating GVHD and inflammatory cytokine storms following allogeneic HCT (26). Consistently, our study demonstrated that treatment with AMD3100 alleviated the severity of GVHD and prolonged survival in human PBMCs implanted mice. Thus, it is reasonable to speculate that AMD3100 plays a protective role in GVHD mice by decreasing the production of inflammatory cytokines. This hypothesis awaits confirmation in future studies.

We also found that the proportion of CCR5-positive cells among peripheral γδ T cells was significantly elevated in recipients with grades II-IV aGVHD compared with those who developed grades 0-I aGVHD (data not shown). This finding was consistent with previous studies that reported a correlation between the expression of CCR5 and aGVHD after alloHCT (27–29). However, the mechanisms by which CCR5 and CXCR4 work are different. CCR5 and its ligands are proinflammatory chemokines that mediate the recruitment of some immune cells and are closely related to the occurrence of various inflammatory diseases (30–32). In contrast, CXCR4 is considered to be a homeostatic factor that is important in immune surveillance (33). In addition, unlike CCR5, which interacts with several types of ligands, CXCR4 has an exclusive ligand known as SDF-1. As reported, the interaction between CXCR4 and SDF-1 mainly regulates the generation, transport, homing and retention of CXCR4-positive blood cells in hematopoietic organs and the accumulation of CXCR4-positive immune cells in inflammatory sites (34–38). Previous studies have shown that SDF-1 correlated with HIV infection, cardiovascular disease and the mobilization of hematopoietic stem and progenitor cells (39–43). Our study demonstrated for the first time that CXCR4 signaling also contributes to the migration of effector T cells and extends to the alloimmune responses that characterize the development of aGVHD.

In summary, aGVHD remains a major obstacle to achieving favorable outcomes following alloHCT. Elucidating the mechanisms by which donor T cells migrate into target organs may represent novel therapeutic opportunities to reduce aGVHD. We demonstrated that γδ T cells with upregulated expression of CXCR4 contributed to CD4 T cell migration into aGVHD target organs after hematopoietic transplantation. Depletion of γδ T cells and treatment with an antagonist of the SDF-1-CXCR4 axis significantly attenuated the severity of aGVHD and prolonged the survival. These findings provide novel insights into how innate-like γδ T cells correlate with alloreactivity. Disrupting SDF-1-CXCR4 interactions might be a possible therapeutic strategy to decrease the incidence of severe aGVHD following alloHCT.

## Data Availability Statement

The raw data supporting the conclusions of this article will be made available by the authors, without undue reservation.

## Ethics Statement

The studies involving human participants were reviewed and approved by Ethics Committee of Peking University Institute of Hematology. The patients/participants provided their written informed consent to participate in this study. The animal study was reviewed and approved by Ethics Committee of Peking University Institute of Hematology.

## Author Contributions

NW and RL designed and performed the research, analyzed data, and wrote the manuscript. SL and HG collected samples and analyzed data. L-PX and X-HZ interpreted data and reviewed the manuscript. JL and X-JH supervised research and revised the manuscript. All authors contributed to the article and approved the submitted version.

## Funding

This study is supported by the National Key Research and Development Program of China (no. 2017YFA0104500) and National Natural Science Foundation of China (no.81770191).

## Conflict of Interest

The authors declare that the research was conducted in the absence of any commercial or financial relationships that could be construed as a potential conflict of interest.

## References

[1] Appelbaum FR. Haematopoietic Cell Transplantation as Immunotherapy. Nature (2001) 411:385–9. 10.1038/35077251 11357147

[2] Zeiser R Blazar BR. Acute Graft-Versus-Host Disease - Biologic Process, Prevention, and Therapy. N Engl J Med (2017) 377:2167–79. 10.1056/NEJMra1609337 PMC6034180 29171820

[3] Malard F Huang XJ Sim JPY. Treatment and Unmet Needs in Steroid-Refractory Acute Graft-Versus-Host Disease. Leukemia (2020) 34:1229–40. 10.1038/s41375-020-0804-2 PMC7192843 32242050

[4] Ferrara JL Levy R Chao NJ. Pathophysiologic Mechanisms of Acute Graft-vs.-Host Disease. Biol Blood Marrow Transplant (1999) 5:347–56. 10.1016/s1083-8791(99)70011-x 10595812

[5] Ferrara JL. Pathogenesis of Acute Graft-Versus-Host Disease: Cytokines and Cellular Effectors. J Hematother Stem Cell Res (2000) 9:299–306. 10.1089/15258160050079407 10894351

[6] Anderson BE Shlomchik WD Shlomchik MJ. Recipient Gammadelta T Cells in Graft-Versus-Host Disease. Blood (2006) 107:3808–9; author reply 3809. 10.1182/blood-2005-11-4586 16627764

[7] Blazar BR Taylor PA Panoskaltsis-Mortari A Barrett TA Bluestone JA Vallera DA. Lethal Murine Graft-Versus-Host Disease Induced by Donor Gamma/Delta Expressing T Cells With Specificity for Host Nonclassical Major Histocompatibility Complex Class Ib Antigens. Blood (1996) 87:827–37. 10.1182/blood.V87.2.827.bloodjournal872827 8555509

[8] Drobyski WR Vodanovic-Jankovic S Klein J. Adoptively Transferred Gamma Delta T Cells Indirectly Regulate Murine Graft-Versus-Host Reactivity Following Donor Leukocyte Infusion Therapy in Mice. J Immunol (2000) 165:1634–40. 10.4049/jimmunol.165.3.1634 10903774

[9] Maeda Y Reddy P Lowler KP Liu C Bishop DK Ferrara JL. Critical Role of Host Gammadelta T Cells in Experimental Acute Graft-Versus-Host Disease. Blood (2005) 106:749–55. 10.1182/blood-2004-10-4087 PMC1895173 15797996

[10] Sackstein R. A Revision of Billingham’s Tenets: The Central Role of Lymphocyte Migration in Acute Graft-Versus-Host Disease. Biol Blood Marrow Transplant (2006) 12:2–8. 10.1016/j.bbmt.2005.09.015 16399577

[11] Springer TA. Traffic Signals for Lymphocyte Recirculation and Leukocyte Emigration: The Multistep Paradigm. Cell (1994) 76:301–14. 10.1016/0092-8674(94)90337-9 7507411

[12] Palmer LA Sale GE Balogun JI Li D Jones D Molldrem JJ. Chemokine Receptor CCR5 Mediates Alloimmune Responses in Graft-Versus-Host Disease. Biol Blood Marrow Transplant (2010) 16:311–9. 10.1016/j.bbmt.2009.12.002 PMC3182111 20025985

[13] Moll NM Ransohoff RM. CXCL12 and CXCR4 in Bone Marrow Physiology. Expert Rev Hematol (2010) 3:315–22. 10.1586/ehm.10.16 21082982

[14] Wang J Tannous BA Poznansky MC Chen H. CXCR4 Antagonist AMD3100 (Plerixafor): From an Impurity to a Therapeutic Agent. Pharmacol Res (2020) 159:105010. 10.1016/j.phrs.2020.105010 32544428

[15] Wang Y Chang YJ Xu LP Liu KY Liu DH Zhang XH. Who Is the Best Donor for a Related HLA Haplotype-Mismatched Transplant? Blood (2014) 124:843–50. 10.1182/blood-2014-03-563130 24916508

[16] Chang YJ Xu LP Wang Y Zhang XH Chen H Chen YH. Controlled, Randomized, Open-Label Trial of Risk-Stratified Corticosteroid Prevention of Acute Graft-Versus-Host Disease After Haploidentical Transplantation. J Clin Oncol (2016) 34:1855–63. 10.1200/jco.2015.63.8817 27091717

[17] Cooke KR Kobzik L Martin TR Brewer J Delmonte J Jr. Crawford JM. An Experimental Model of Idiopathic Pneumonia Syndrome After Bone Marrow Transplantation: I. The Roles of Minor H Antigens and Endotoxin. Blood (1996) 88:3230–9. 10.1182/blood.V88.8.3230.bloodjournal8883230 8963063

[18] Paul S Singh AK Shilpi Lal G. Phenotypic and Functional Plasticity of Gamma-Delta (Gammadelta) T Cells in Inflammation and Tolerance. Int Rev Immunol (2014) 33:537–58. 10.3109/08830185.2013.863306 24354324

[19] Zheng J Liu Y Lau YL Tu W. Gammadelta-T Cells: An Unpolished Sword in Human Anti-Infection Immunity. Cell Mol Immunol (2013) 10:50–7. 10.1038/cmi.2012.43 PMC4003172 23064104

[20] Aggarwal R Lu J Kanji S Das M Joseph M Lustberg MB. Human Vgamma2Vdelta2 T Cells Limit Breast Cancer Growth by Modulating Cell Survival-, Apoptosis-Related Molecules and Microenvironment in Tumors. Int J Cancer (2013) 133:2133–44. 10.1002/ijc.28217 PMC3939063 23595559

[21] Deniger DC Maiti SN Mi T Switzer KC Ramachandran V Hurton LV. Activating and Propagating Polyclonal Gamma Delta T Cells With Broad Specificity for Malignancies. Clin Cancer Res (2014) 20:5708–19. 10.1158/1078-0432.CCR-13-3451 PMC4233015 24833662

[22] Hu Y Cui Q Luo C Luo Y Shi J Huang H. A Promising Sword of Tomorrow: Human Gammadelta T Cell Strategies Reconcile Allo-HSCT Complications. Blood Rev (2016) 30:179–88. 10.1016/j.blre.2015.11.002 26654098

[23] Pabst C Schirutschke H Ehninger G Bornhauser M Platzbecker U. The Graft Content of Donor T Cells Expressing Gamma Delta TCR+ and CD4+foxp3+ Predicts the Risk of Acute Graft Versus Host Disease After Transplantation of Allogeneic Peripheral Blood Stem Cells From Unrelated Donors. Clin Cancer Res (2007) 13:2916–22. 10.1158/1078-0432.CCR-06-2602 17504991

[24] Kawanishi Y Passweg J Drobyski WR Rowlings P Cook-Craig A Casper J. Effect of T Cell Subset Dose on Outcome of T Cell-Depleted Bone Marrow Transplantation. Bone Marrow Transplant (1997) 19:1069–77. 10.1038/sj.bmt.1700807 9193748

[25] Kang Y Chen BJ Deoliveira D Mito J Chao NJ. Selective Enhancement of Donor Hematopoietic Cell Engraftment by the CXCR4 Antagonist AMD3100 in a Mouse Transplantation Model. PloS One (2010) 5:e11316. 10.1371/journal.pone.0011316 20596257 PMC2893128

[26] Green MM Chao N Chhabra S Corbet K Gasparetto C Horwitz A. Plerixafor (a CXCR4 Antagonist) Following Myeloablative Allogeneic Hematopoietic Stem Cell Transplantation Enhances Hematopoietic Recovery. J Hematol Oncol (2016) 9:71. 10.1186/s13045-016-0301-2 27535663 PMC4989381

[27] Ma Q Gooley TA Storb RF. CCR5 Expression on Cells From HLA-Matched Unrelated Marrow Donors and Graft-Versus-Host Disease. Biol Blood Marrow Transplant (2010) 16:132–3. 10.1016/j.bbmt.2009.05.017 PMC2804929 20053336

[28] Murai M Yoneyama H Harada A Yi Z Vestergaard C Guo B. Active Participation of CCR5(+)CD8(+) T Lymphocytes in the Pathogenesis of Liver Injury in Graft-Versus-Host Disease. J Clin Invest (1999) 104:49–57. 10.1172/JCI6642 10393698 PMC408408

[29] Welniak LA Wang Z Sun K Kuziel W Anver MR Blazar BR. An Absence of CCR5 on Donor Cells Results in Acceleration of Acute Graft-vs-Host Disease. Exp Hematol (2004) 32:318–24. 10.1016/j.exphem.2003.12.003 15003318

[30] Lan YY Wang YQ Liu Y. CCR5 Silencing Reduces Inflammatory Response, Inhibits Viability, and Promotes Apoptosis of Synovial Cells in Rat Models of Rheumatoid Arthritis Through the MAPK Signaling Pathway. J Cell Physiol (2019) 234:18748–62. 10.1002/jcp.28514 31066041

[31] Ye X Liu S Hu M Song Y Huang H Zhong Y. CCR5 Expression in Inflammatory Bowel Disease and Its Correlation With Inflammatory Cells and Beta-Arrestin2 Expression. Scand J Gastroenterol (2017) 52:551–7. 10.1080/00365521.2017.1281435 28140695

[32] Ikebuchi R Fujimoto M Nakanishi Y Okuyama H Moriya T Kusumoto Y. Functional Phenotypic Diversity of Regulatory T Cells Remaining in Inflamed Skin. Front Immunol (2019) 10:1098. 10.3389/fimmu.2019.01098 31156643 PMC6534040

[33] Demmer O Dijkgraaf I Schumacher U Marinelli L Cosconati S Gourni E. Design, Synthesis, and Functionalization of Dimeric Peptides Targeting Chemokine Receptor CXCR4. J Med Chem (2011) 54:7648–62. 10.1021/jm2009716 21905730

[34] Galli E Hartmann FJ Schreiner B Ingelfinger F Arvaniti E Diebold M. GM-CSF and CXCR4 Define a T Helper Cell Signature in Multiple Sclerosis. Nat Med (2019) 25:1290–300. 10.1038/s41591-019-0521-4 PMC6689469 31332391

[35] Levesque JP Hendy J Takamatsu Y Simmons PJ Bendall LJ. Disruption of the CXCR4/CXCL12 Chemotactic Interaction During Hematopoietic Stem Cell Mobilization Induced by GCSF or Cyclophosphamide. J Clin Invest (2003) 111:187–96. 10.1172/JCI15994 PMC151860 12531874

[36] Zhang Y Foudi A Geay JF Berthebaud M Buet D Jarrier P. Intracellular Localization and Constitutive Endocytosis of CXCR4 in Human CD34+ Hematopoietic Progenitor Cells. Stem Cells (2004) 22:1015–29. 10.1634/stemcells.22-6-1015 15536192

[37] Ngamsri KC Jans C Putri RA Schindler K Gamper-Tsigaras J Eggstein C. Inhibition of CXCR4 and CXCR7 Is Protective in Acute Peritoneal Inflammation. Front Immunol (2020) 11:407. 10.3389/fimmu.2020.00407 32210974 PMC7076176

[38] Isles HM Herman KD Robertson AL Loynes CA Prince LR Elks PM. The CXCL12/CXCR4 Signaling Axis Retains Neutrophils At Inflammatory Sites in Zebrafish. Front Immunol (2019) 10:1784. 10.3389/fimmu.2019.01784 31417560 PMC6684839

[39] Oberlin E Amara A Bachelerie F Bessia C Virelizier JL Arenzana-Seisdedos F. The CXC Chemokine SDF-1 Is the Ligand for LESTR/fusin and Prevents Infection by T-Cell-Line-Adapted HIV-1. Nature (1996) 382:833–5. 10.1038/382833a0 8752281

[40] Zhong J Rajagopalan S. Dipeptidyl Peptidase-4 Regulation of SDF-1/CXCR4 Axis: Implications for Cardiovascular Disease. Front Immunol (2015) 6:477. 10.3389/fimmu.2015.00477 26441982 PMC4585326

[41] van der Vorst EP Doring Y Weber C. MIF and CXCL12 in Cardiovascular Diseases: Functional Differences and Similarities. Front Immunol (2015) 6:373. 10.3389/fimmu.2015.00373 26257740 PMC4508925

[42] Lataillade JJ Clay D Dupuy C Rigal S Jasmin C Bourin P. Chemokine SDF-1 Enhances Circulating CD34(+) Cell Proliferation in Synergy With Cytokines: Possible Role in Progenitor Survival. Blood (2000) 95:756–68. 10.1182/blood.V95.3.756 10648383

[43] Richter R Jochheim-Richter A Ciuculescu F Kollar K Seifried E Forssmann U. Identification and Characterization of Circulating Variants of CXCL12 From Human Plasma: Effects on Chemotaxis and Mobilization of Hematopoietic Stem and Progenitor Cells. Stem Cells Dev (2014) 23:1959–74. 10.1089/scd.2013.0524 PMC4120931 24708339

