# Supplementary material for: γδ T Cells May Aggravate Acute Graft-Versus-Host Disease Through CXCR4 Signaling After Allogeneic Hematopoietic Transplantation
Source: Front Immunol. 2021 Jul 14;12:687961. doi: 10.3389/fimmu.2021.687961 (PMC8316995; doi:10.3389/fimmu.2021.687961)
Supplement: Supplementary file 1 [file DataSheet_1.pdf]

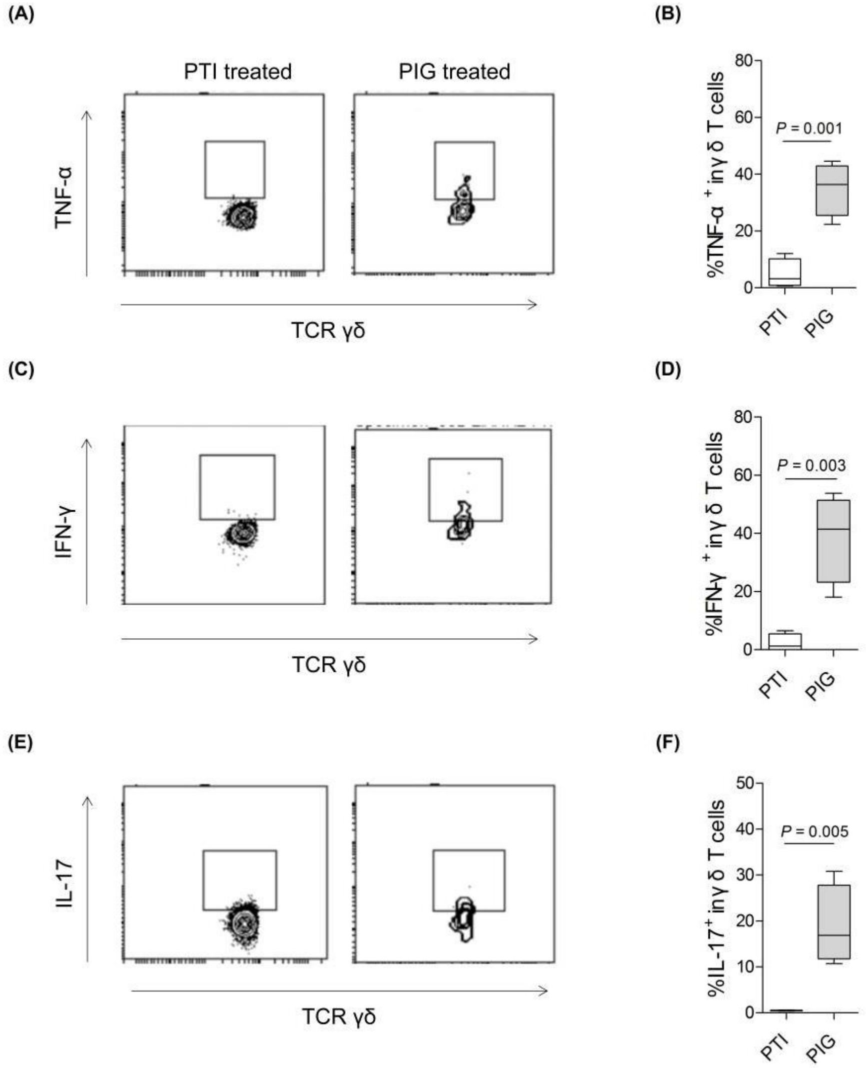

Supplementary Figure 1. Intracellular cytokine staining on γδ T cells.

PBMCs were freshly isolated from healthy donors and cultured in 1640 medium plus 10% FBS for 3 days. Then cells were treated with Protein Transport Inhibitor Cocktail (PTI, 500x, eBioscience, USA) or Cell Stimulation Cocktail (plus protein transport inhibitors) (PIG, 500x, eBioscience, USA) for 5h followed by being stained with antibodies against γδ TCR (A–F), TNF-α (A, B), IFN-γ (C, D) and IL-17 (E, F). Data are expressed as the mean ± SD of triplicate cultures. *P* values are shown on the graphs.

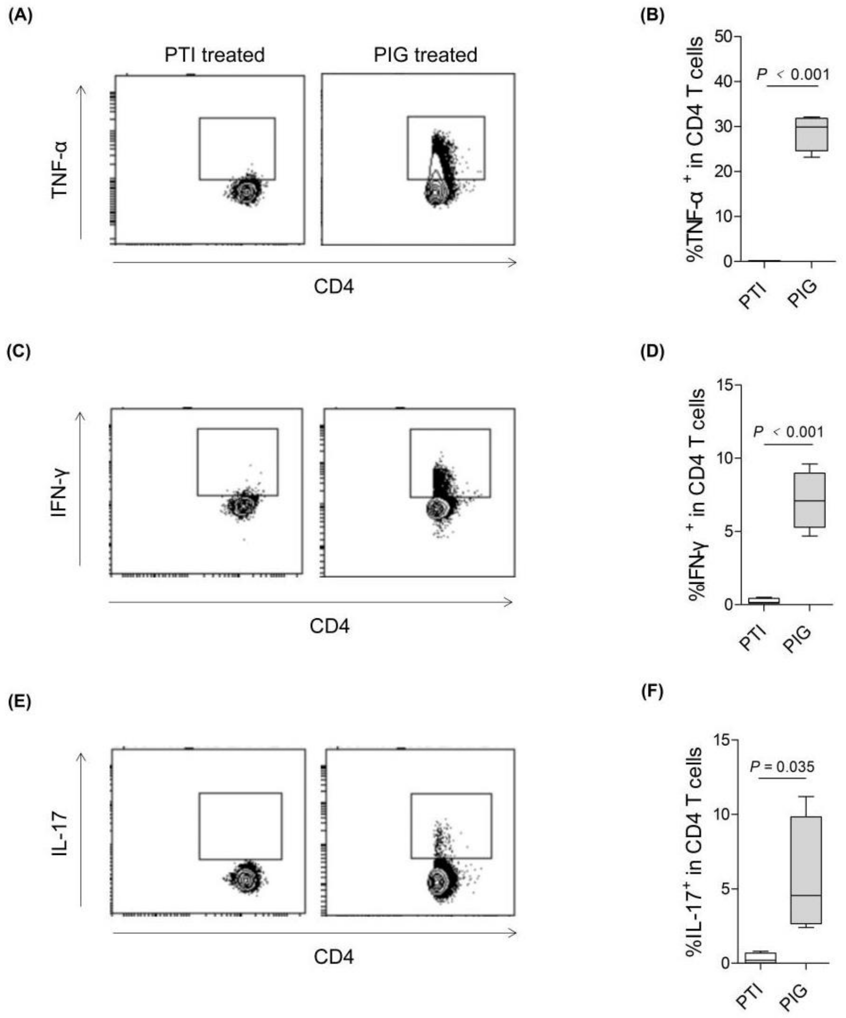

Supplementary Figure 2. Intracellular cytokine staining on CD4^{+} T cells.

Intracellular cytokine staining with antibodies against CD4 (A–F), TNF-α (A, B), IFN-γ (C, D) and IL-17 (E, F) was performed on PBMCs treated with Protein Transport Inhibitor Cocktail (PTI, 500x, eBioscience, USA) or Cell Stimulation Cocktail (plus protein transport inhibitors) (PIG, 500x, eBioscience, USA) for 5h after cells have been cultured in 1640 medium plus 10% FBS for 3 days. Data are expressed as the mean ± SD of triplicate cultures. *P* values are shown on the graphs.

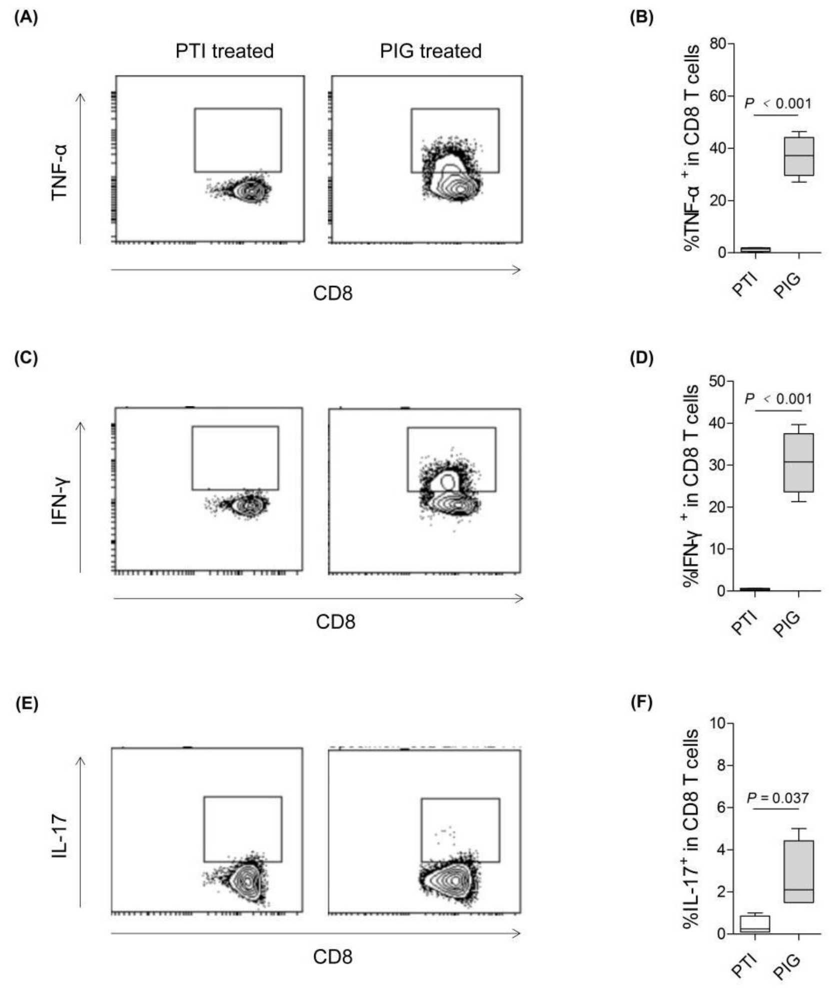

Supplementary Figure 3. Intracellular cytokine staining on CD8$^+$ T cells.

Following being cultured in 1640 medium plus 10% FBS for 3 days, PBMCs were treated with Protein Transport Inhibitor Cocktail (PTI, 500x, eBioscience, USA) or Cell Stimulation Cocktail (plus protein transport inhibitors) (PIG, 500x, eBioscience, USA) for 5h and evaluated using flow cytometry for intracellular cytokine: CD8 (A–F), TNF-α (A, B), IFN-γ (C, D) and IL-17 (E, F). Data are expressed as the mean ± SD of triplicate cultures. *P* values are shown on the graphs.

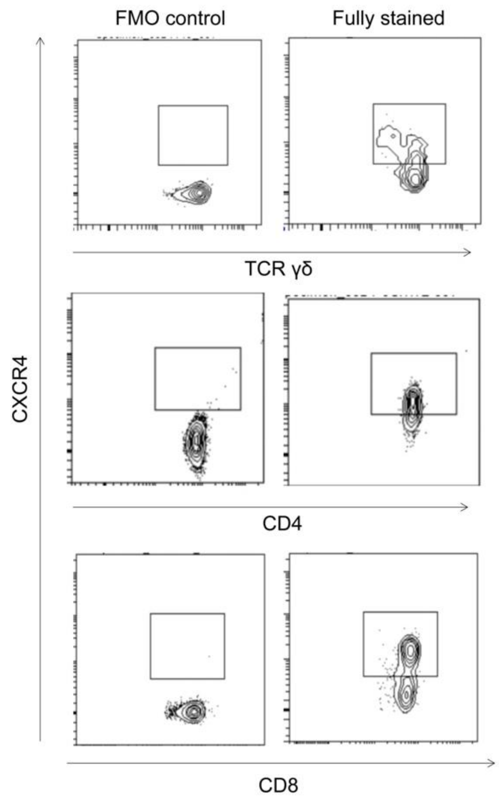

Supplementary Figure 4. Gating strategy for identifying CXCR4-expressing T cell subsets.

The gating strategy for the positive populations shown in Figure 5A, 5C & 5E were based on blank (cells without staining) and Fluorescence Minus One (FMO) control. In the fully stained sample, fluorochrome-conjugated antibodies specific for human CD3, TCRαβ, CD4, CD8, CXCR4 were applied to detect CXCR4 expression on γδ T, CD4 T and CD8 T cells. FMO control was labeled CD3, TCRαβ, CD4, CD8 antibodies, but not CXCR4.
